# Ethylene Improves Root System Development under Cadmium Stress by Modulating Superoxide Anion Concentration in *Arabidopsis thaliana*

**DOI:** 10.3389/fpls.2017.00253

**Published:** 2017-02-24

**Authors:** Ann Abozeid, Zuojia Ying, Yingchao Lin, Jia Liu, Zhonghua Zhang, Zhonghua Tang

**Affiliations:** ^1^Key Laboratory of Plant Ecology, Northeast Forestry UniversityHarbin, China; ^2^Botany Department, Faculty of Science, Menoufia UniversityShibin El Kom, Egypt; ^3^The College of Landscape, Northeast Forestry UniversityHarbin, China; ^4^Guizhou Academy of Tobacco ResearchGuiyang, China

**Keywords:** ethylene, Cd stress, superoxide, programmed cell death, root system, *Arabidopsis thaliana*

## Abstract

This work aims at identifying the effects of ethylene on the response of *Arabidopsis thaliana* root system to cadmium chloride (CdCl_2_) stress. Two ethylene-insensitive mutants, *ein2-5* and *ein3-1eil1-1*, were subjected to (25, 50, 75, and 100 μM) CdCl_2_ concentrations, from which 75 μM concentration decreased root growth by 40% compared with wild type Col-0 as a control. Ethylene biosynthesis increased in response to CdCl_2_ treatment. The length of primary root and root tip in *ein2-5* and *ein3-1eil1-1* decreased compared with wild type after CdCl_2_ treatment, suggesting that ethylene play a role in root system response to Cd stress. The superoxide concentration in roots of *ein2-5* and *ein3-1eil1-1* was greater than in wild type seedlings under Cd stress. Application of exogenous 1-aminocyclopropane-1-carboxylic acid (ACC) (a precursor of ethylene biosynthesis) in different concentrations (0.01, 0.05 and 0.5 μM) decreased superoxide accumulation in Col-0 root tips and increased the activities of superoxide dismutase (SOD) isoenzymes under Cd stress. This result was reversed with 5 μM of aminoisobutyric acid AIB (an inhibitor of ethylene biosynthesis). Moreover, it was accompanied by increase in lateral roots number and root hairs length, indicating the essential role of ethylene in modulating root system development by controlling superoxide accumulation through SOD isoenzymes activities. The suppressed Cd-induced superoxide accumulation in wild type plants decreased the occurrence of cells death while programmed cell death (PCD) was initiated in the root tip zone, altering root morphogenesis (decreased primary root length, more lateral roots and root hairs) to minimize the damage caused by Cd stress, whereas this response was absent in the *ein2-5* and *ein3-1eil1-1* seedlings. Hence, ethylene has a role in modulating root morphogenesis during CdCl_2_ stress in *A. thaliana* by increasing the activity of SOD isoenzymes to control superoxide accumulation.

## Introduction

Heavy metal toxicity in soil was once limited to localities affected by mining, heavy industry or derived from natural mineral outcrops. Metal toxicity not only reduces crop productivity but also threatens the food chain (Poschenrieder and Barceló, 1999). Among the common heavy metal pollutants, cadmium (Cd) is perhaps one of the most aggressive and persistent and is also the most susceptible to accumulation through inappropriate agricultural practices (Choppala et al., 2014; Asgher et al., 2015; Wahid and Khaliq, 2015). The major Cd input into agricultural soils is the phosphorus fertilizers (Singh and Agrawal, 2007). Plants affected by Cd showed impaired photosynthesis (Burzynski and Klobus, 2004; Andresen et al., 2016), altered mineral nutrition and carbohydrate metabolism (Azevedo et al., 2005; Santos et al., 2010) and water imbalance (Sandalio et al., 2001). Cadmium is a non-essential element but is readily absorbed by roots and transported to the aerial parts of plants. Cadmium concentrations of 1–5 μM in the soil solution are sufficient to retard root growth: presumably such toxicity occurs because Cd can replace essential elements that play key roles at the active sites of enzymes, thus affecting many aspects of plant growth and development (Sofo et al., 2013; Vitti et al., 2013).

The plant hormone ethylene is involved in many aspects of the plant life cycle, including seed germination and root hair development (Johnson and Ecker, 1998). It is considered as a (stress hormone) for modulating a diverse array of defense responses (Montero-Palmero et al., 2014; Pan et al., 2016). Thus, ethylene is highly expected to play a role in plant response to Cd stress.

Ethylene acts as a regulator of stress-related morphological responses such as primary and secondary root growth (Parlanti et al., 2011). Interestingly, ethylene remodels the root system architecture to increase Pi uptake in responses to low phosphate (Pi) (Nagarajan and Smith, 2012). This indicates the role of ethylene in root development in relations to heavy metal stress.

Ethylene is derived from methionine, and the first committed step in this pathway is the conversion of *S*-adenosyl methionine to 1-aminocyclopropane-1-carboxylate (ACC), which is performed by the enzyme ACC synthase (ACS). ACC is then oxidized by ACC oxidase to form ethylene, CO_2_ and cyanide. During these processes, ACS-catalyzed ACC synthesis is generally the rate-limiting step for ethylene production (Skottke et al., 2011). The ACS isozymes are encoded for by a large gene family, among which phosphorylation of the type 1 isozymes ACS2 and ACS6 by stress-responsive MAPKs results in increased ethylene synthesis (Han et al., 2010; Skottke et al., 2011).

In *Arabidopsis*, ethylene signaling is first perceived by a family of five receptors that are located at the Golgi and endoplasmic reticulum (ER) membranes (Dong et al., 2010). Ethylene binding is proposed to inhibit receptor function, and CONSTITUTIVE TRIPLE RESPONSE 1 (CTR1) is proposed to be activated by the unoccupied receptor via a physiological interaction (Guo and Ecker, 2003), resulting in the activation of ETHYLENE INSENSITIVE 2 (EIN2). EIN2 plays a central role in the ethylene signaling transduction pathway, and the *ein2* mutant, is completely insensitive to ethylene at the morphological, physiological and molecule levels in *Arabidopsis* (Shibuya et al., 2004). The ethylene signaling pathway then continues to the nuclear transcriptional factors EIN3 and EIN3-like (EIL). In *Arabidopsis*, there are six members in the EIN3 family, of which EIN3 and its close homolog EIL1 have been widely studied (An et al., 2010; Lingam et al., 2011; Zhu et al., 2011). Mutants in EIN3 and EIL1 have weak ethylene insensitivity (Chao et al., 1997; Binder et al., 2007). Interestingly, *ein3 eil1* double mutants display complete ethylene insensitivity in all known ethylene responses, including the triple response and pathogen resistance (Guo and Ecker, 2003). Subsequently, these primary transcription factors activate other secondary transcription factors, such as the ETHYLENE RESPONSE FACTORs (ERFs), thereby regulating the expression of genes that are involved in the response to ethylene (Guo and Ecker, 2004; Kendrick and Chang, 2008). It has been demonstrated that the expression of ERF1 can be activated rapidly by ethylene (Lorenzo et al., 2003).

The production of ethylene is tightly regulated by internal signals during development and by responses to environmental stimuli from biotic and abiotic stresses, such as wounding, ozone, chilling or freezing (Wang et al., 2002; Yoo et al., 2009). Cd induces ethylene biosynthesis in the wild-type of *Arabidopsis thaliana* (Schellingen et al., 2014). Under Cd stress, the production of ethylene increases drastically in the root tip (Arteca and Arteca, 2007), the region where superoxide is predominantly localized (Dunand et al., 2007), which led us to investigate the connections between ethylene biosynthesis and superoxide production in the root tip. Cd stress is also related to the increase of ROS (Xu et al., 2010a), the oxidative damage of ROS requires the action of antioxidative enzymes, including superoxide dismutase (SOD), which can convert superoxide radicals into hydrogen peroxide, water and oxygen. SOD activity increased by the application of exogenous ACC and ethylene decrease the concentration of hydrogen peroxide under abiotic stress (Lin et al., 2012, 2013a,b). This better support our hypothesis that ethylene regulate superoxide accumulation through SOD activity.

Programmed cell death (PCD) is a functional term used to describe cell death that eliminates harmful cells during the life cycle of multicellular organisms. Increasing evidence suggests that diverse abiotic stresses, such as salt, drought, nutrient deficiency and heavy metal toxicity, can induce PCD in plant root tips (Liu et al., 2009; Duan et al., 2010; Xu et al., 2010b; Demidchik et al., 2014). Under drought and long-term zinc exposure, the PCD program is activated in the root apical meristematic zone, so apical root dominance is removed. Thus, the root system architecture may be remodel allowing adaptation to the stressful environment (Duan et al., 2010; Xu et al., 2010b). This led us to expect that PCD might be the mechanism by which the plant responds to the damage caused by Cd stress especially because reactive oxygen species (ROS) accumulation may trigger the PCD process in stressed plants (Harding and Roberts, 1998; Chae and Lee, 2001). However, the relation between production of superoxide and the progress of PCD in root tips under Cd stress is still unknown.

So our hypothesis suggests that ethylene modulates root system by decreasing superoxide accumulations through decreasing NADPH oxidase activity and increasing SOD enzymes activity. Ethylene-induced suppression of superoxide accumulation modulates the root development through decreasing cell death (damaging effect of superoxide accumulation) and initiating PCD.

The aim of this study is to highlight the role of ethylene in response to the damage effect caused by Cd stress through its effects on superoxide accumulation by increasing the SOD activity, and to show the relation between the progression of cell death and PCD in root tips and the mechanism by which ethylene in minimize the damage effect caused by Cd stress, to propose a schematic model elucidating this mechanism by which the plant responses to Cd stress through inducing ethylene biosynthesis to modulate the root system development.

## Materials and Methods

### Plant Material and Growth Conditions

Seedlings of the following lines were used in this study: *Arabidopsis thaliana* ecotype Columbia-0 (Col-0) and the ethylene-insensitive mutants *ein2-5* (Alonso et al., 1999) and *ein3-1eil1-1* (Alonso et al., 2003) in the Col-0 background. All seeds were surface-sterilized by incubation in 70% ethanol containing 0.05% Triton X-100 (Solar Bio, Beijing, China) for 10 min, rinsed thoroughly with ethanol for 1 min and then washed with sterilized water. The sterilized seeds were sown on agar plates containing a MS basal salt mixture (Sigma–Aldrich, St. Louis, MO, USA), 1% sucrose and 0.8% agar (pH 5.7). The plates were maintained at 4°C in the dark for 48 h to enhance the germination process and then placed vertically at 23°C under a light intensity of 200 μml m^-2^ s^-1^ (16/8 h light cycle).

### Treatments

To investigate the effects of Cd stress on root growth, 4-day-old seedlings cultivated in control agar plates were transferred to freshly prepared medium supplemented by various concentration of CdCl_2_ (25, 50, 75, and 100 μM) as indicated (Cao et al., 2009; Radeva et al., 2010) for 2 and 4 days. Preliminary experiments with seedling grown with various CdCl_2_ concentrations showed that 75 μM CdCl_2_ decreased root growth by about 40% and so this concentration was used to compare the effects of Cd stress between the wild type and the ethylene-insensitive mutants.

To investigate the effects of exogenous ACC on the accumulation of superoxide in the root tips of the Col-0 plants under Cd stress. Four-day-old seedlings were transferred to various pretreated agar plates with 75 μM CdCl_2_ or 75 μM CdCl_2_ plus various concentrations of ACC (0.01, 0.05, and 0.5 μM, a precursor of ethylene biosynthesis) with or without 5 μM AIB (an inhibitor of ethylene biosynthesis), as indicated, for 4 days.

All the experiments were repeated three times with three replicates of each treatment unless otherwise noted.

### Quantitative RT-PCR Analysis of Gene Expression

To investigate whether ethylene biosynthesis and signaling were involved in the plant response to Cd stress, the relative expression of genes encoding for ethylene biosynthesis, and of perception and signaling proteins were measured in seedlings that were treated with different concentrations of CdCl_2_ for 4 days. Total RNA was isolated from 100 seedling roots using TRIzol solution (Invitrogen, Carlsbad, CA, USA). Two micrograms of total RNA was used for first-strand cDNA synthesis using RevertAid Reverse Transcriptase and Oligo d(T)primers (Takara, Dalian, China). The cDNA yield was measured according to the PCR signal generated from the internal standard, with the housekeeping gene *Actin 2* used as the internal control. The primers that were used in the quantitative RT-PCR are listed in **Table 1** (Liu et al., 2010; Lin et al., 2013b; Li et al., 2014).

**Table 1:** The primers used in the quantitative RT-PCR.

Gene	Gene Bank accession no.	Primers
*ACTIN2*	At3g18780	F:5′- TGTGCCAATCTACGAGGGT-3′ R:5′- GCTGGTCTTTGAGGTTTCC-3′
*ACS2*	At1g01480	F:5′- AGGCAATTGCACATTTCATGG-3′ R:5′- CTGTCCGCCACCTCAAGTCT-3′
*EIN2*	At5g03280	F:5′- TGCGCATGCACTTAACCTTTT-3′ R:5′- TGACTCAGCAAGACGCCAGA-3′
*EIN3*	At3g20770	F:5′- AGGCAGAGACCTTTTTCTTCATCA-3′ R:5′- CAGGCTCAGCTTGTGGAACA-3′

Quantitative RT-PCR was performed using a RealMasterMix kit (Tiangen, Beijing, China) with 25 cycles as follows: 94°C for 30 s, 58°C for 30 s and 72°C for 30 s, followed by 72°C for 10 min gene expression quantifications was performed using the relative 2^-ΔΔCt^ method (Livak and Schmittgen, 2001). All experiments were performed in triplicate for each treatment.

### GUS Staining

To investigate the mechanism by which CdCl_2_ affects the synthesis and distribution of ethylene, the relative expression of the ethylene reporter construct, EBS::GUS, in which the GUS reporter gene is driven by a synthetic EIN3-responsive promoter, was tested. GUS staining was as described by He et al. (2011). Four-day-old seedlings were transferred to agar plates supplemented with 75 μM CdCl_2_ for 4 days, then were collected and washed with staining buffer without X-Gluc and stained with GUS staining buffer (50 mM sodium phosphate buffer, pH 7.0, 10 mM Na_2_EDTA, 0.5 mM K_4_[Fe(CN)_6_]⋅3H2O, 0.5 mM K_3_[Fe(CN)_6_], 0.1% Triton X-100, and 1 mg/mL *X*-Gluc). Ethanol (70%) was used to terminate the staining reaction, and the seedlings were mounted on slides in 50 μL Hoyer’s solution (chloral hydrate: water: glycerol; 8:3:1; w/v/v) and examined and photographed with a DM 4000B stereomicroscope (Leica, Germany).

### Morphometric Analysis

To further test the involvement of ethylene signaling in regulating the plant response to Cd stress, two ethylene-insensitive mutants (*ein2-5* and *ein3-1eil1-1*) together with the wild type plant were subjected to Cd stress with or without ACC. The length of the whole primary root of the seedlings were measured either directly with a ruler or using NIS Elements software (Nikon, Japan) from digital images captured with a Nikon camera. The root tip length was also determined in the same way as the primary root. The number of the lateral roots was counted. The number of root hairs in a 2 mm section at the appropriate midpoint of the root was counted under a dissecting microscope. Root hairs from the photographs were measured with a ruler, and their length in μm was determined by comparison with an ocular micrometer photograph at the same magnification. All experiments for phenotypic analysis were performed at least three times, and the data represent one independent experiment.

### Cadmium Analysis

The samples were rinsed twice with tap water then with de-ionized water before air drying is used to remove dirt and salt from root surface. High purity deionized water from a Millipore water purification system (Bedford, NY, USA) was used throughout our study. The root materials were dried at 80°C to a constant weight. The dried tissues were weighed and digested in HNO3 (100%) using the heat block (Shah et al., 2014; Olowu et al., 2015). The metal concentration was determined by inductively coupled plasma mass spectrometry (ICP-MS). Blanks (only HNO3) were analyzed for reference purposes. The cadmium standard solutions for ICP-MS (TraceCERT^®^, Sigma–Aldrich) were produced according to the ISO Guide 34 in the analysis of Cd concentrations. External calibration was performed using a five-point analytical curve, prepared by diluting the individual cadmium standards with 5.0% (v/v) HNO3.

Bioaccumulation factor (Bf) was calculated to measure plant uptake of Cd:

$$\text{Bf}\;\text{=}\;\frac{\text{Cd}\;\text{concentration}\;\text{in}\;\text{tissues}\;({\text{gkg}}^{\text{-1}})}{\text{Cd}\;\text{concentration}\;\text{in}\;\text{solution}\;({\text{gL}}^{\text{-1}})}.$$

### Measurement of Superoxide Accumulation in Root Tips by (NBT) Staining

Four-days-old wild-type seedlings cultivated on MS agar plates were transferred to agar plates supplemented with 75 μM CdCl_2_ or 75 μM CdCl_2_ plus various concentrations of ACC (0.01, 0.05, and 0.5 μM, a precursor of ethylene biosynthesis) with or without 5 μM AIB (an inhibitor of ethylene biosynthesis). Superoxide within the root tip was detected by nitroblue tetrazolium (NBT) staining which is used to detect O_2_^-^ production, as well as other compounds (such as ascorbate) as described by Hernández et al. (2001). Seedlings were collected, and the roots were immersed in a 0.1% solution of NBT in 50 mM K–phosphate buffer (pH 6.4), containing 10 mM Na-azide in the absence of light. Seedlings were then transferred to distilled water to stop the reaction. The roots were observed and photographed using a DM 4000B Leica stereomicroscope equipped with a DC300F Nikon camera. The NIH ImageJ software was used to assess the mean staining intensity of the elongation and meristematic zones (0, white; 255, black) following Dunand et al. (2007). At least 20 individual roots were analyzed for each genotype and treatment, and one representative image was selected for the figure.

### NADPH-Dependent O_2_^⋅-^ Determination

The determination of the NADPH dependent O_2_^-^ generating activity in isolated plasma membrane vesicles was carried out according to Quartacci et al. (2001) and Van Gestelen et al. (1997), by measuring the rate of SOD inhibitable reduction of NBT using NADPH as electron donor. The reaction mixture consisted of 50 mM TRIS HCl buffer, pH 7.5, 0.25 M sucrose, 0.1 mM NBT, and 50–100 μg proteins. After 1 min preincubation the reaction started by the addition of 0.1 mM NADPH and the absorbance changes at 530 nm were followed for 5 min. Rates of O_2_^-^ generation were calculated using an extinction coefficient of 12.8 mM^-1^cm^-1^.

### Measurement of SOD and Its Three Isoenzymes Activities

To investigate whether ethylene reduced Cd stress-induced superoxide accumulation through the SOD pathway, 4-day-old seedlings were transferred to various agar plates for 4 days, and the activities of SOD and its three isoenzymes (Cu/Zn-SOD, Fe-SOD and Mn-SOD) were measured according to the method of Yu and Rengel (1999) with minor modifications. Frozen root samples (0.05 g) were weighed and homogenized on ice for 2 min in 5 ml of homogenizing solution containing 50 mm HEPES buffer and 0.1 mm Na_2_EDTA (pH 7.6). The homogenate was centrifuged at 15000 *g* for 15 min at 4°C to produce the crude extract for SOD assays. SOD activity was assayed by monitoring the inhibition of the photochemical reduction of NBT. For the total SOD assay, a 5-ml reaction mixture containing 50 mM HEPES (pH 7.6), 0.1 mM EDTA, 50 mM Na_2_CO_3_ (pH 10.4), 13 mM methionine, 0.025% (w/v) Triton X-100, 75 μM NBT, 2 μM riboflavin and an appropriate aliquot of enzyme extract was utilized. The reaction mixtures were illuminated for 15 min at a light intensity of 350 μmol⋅m^-2^⋅s^-1^. One unit of SOD activity was defined as the amount of enzyme required to cause a 50% inhibition in the reduction of NBT, as monitored at 560 nm. The activities of different SOD forms were identified by adding KCN and/or H_2_O_2_ to the reaction mixture (Giannopolitis and Ries, 1977). KCN inhibits Cu/Zn-SOD but does not affect Mn-SOD or Fe-SOD, whereas H_2_O_2_ inactivates Cu/Zn-SOD and Fe-SOD but not Mn-SOD. In addition, peroxidases might interfere with the SOD assay in the presence of exogenous H_2_O_2_ (Yu et al., 1998). After the extensive preliminary testing of a range of concentrations, KCN (at final concentration of 3 mM) was added to the reaction mixture before the addition of H_2_O_2_ (5 mM final concentration) to eliminate the interference of peroxidase and catalase enzymes (Chen and Asada, 1989). Mn-SOD activity was determined in the presence of both 3 mM KCN and 5 mM H_2_O_2_. Fe-SOD activity was obtained by subtracting the Mn-SOD activity from the activity in the presence of 3 mM KCN, and Cu/Zn-SOD activity was calculated from the differences between the total activity and that of Mn-SOD and Fe-SOD. Identical reaction mixtures that had not been illuminated were used to correct for background absorbance.

### Detection of Cell Death in Meristimatic and Elongation Zones under Cd Stress with Trypan Blue and Propidium Iodide (PI) Staining

To assess cell death in the root tips under Cd stress, the roots were immersed in 4 mg ml^-1^ Trypan blue solution (Sigma–Aldrich, Saint Louis, MO, USA) for 15 min at room temperature and then washed with distilled water three times. The samples were then observed by light microscopy, and pictures were taken as detailed earlier.

For PI staining, the seedlings were immersed in the PI (Sigma–Aldrich, St. Louis, MO, USA) solution (final concentration of 1 μg ml^-1^) for 10 min at room temperature in the dark and then washed with phosphate buffer solution (PBS) (pH 7.4) three times. The samples were then examined using a DM 4000B fluorescence microscope (Leica, Germany) with an excitation wavelength of 546 nm. Both experiments were repeated three times. For each treatment and genotype, at least 20 roots were analyzed for both stains, and one representative image was selected for the figure.

### Terminal Deoxynucleotidyl Transferase-Mediated dUTP Nick End Labeling (TUNEL) and 4, 6-Diamidino -2-Phenylindole (DAPI) Assay

Cadmium-induced cell death in root tips can occur through either necrosis or PCD. To examine whether PCD is involved in the cell death at the root tips, we investigated the chromatin condensation and the internucleosomal fragmentation of the DNA by DAPI staining and the TUNEL assay, respectively. To detect cell apoptosis caused by Cd stress, an *In Situ* Cell Death Detection Kit, AP (Roche, Germany) was used. The TUNEL assay was performed according to the manufacturer’s instructions with a few modifications. In brief, whole seedlings were fixed in 4% paraformaldehyde in PBS (pH 7.4) for 20 min (20°C). After washing the samples for 30 min with PBS, the samples were incubated in permeabilization solution (0.1% Triton X-100, 0.1% sodium citrate) for 2 min (4°C), followed by two washes with PBS. Fifty microliters of TUNEL reaction mixture was then added to the sample, and it was incubated for 60 min (37°C) in a humidified atmosphere in the dark. After being rinsed three times with PBS, the samples were analyzed under a DM 4000B fluorescence microscope (Leica, Germany) with a 488 nm excitation line and a 530 nm emission filter.

DAPI staining was performed by fixing roots in a solution of ethanol and acetic acid (3:1) for 1 h. The roots were then washed twice with PBS (pH 7.4) and stained in PBS containing 1 μg/ml of DAPI for 20 min at room temperature. After two PBS washes, the samples were analyzed under a DM 4000B fluorescence microscope (Leica, Germany) with a UV light filter, and the images were captured immediately. Both experiments were repeated three times. For each treatment and genotype, at least 20 roots were analyzed for each experiment, and one representative image was selected for the figure.

### Statistical Analyses

For all experiments, the data were statistically analyzed using SPSS 17.0 (SPSS, Chicago, IL, USA). One-way analysis of variance with a Duncan *post hoc* test was used to test the differences at a 0.05 level for the primary root length and distance to the first root hair. The data presented here are the means with standard error (SE).

NADPH oxidase and SOD enzymes activities values were used for the calculation of abundance ratios between groups and for statistical evaluation by Student’s *t*-test (*P* = 0.05).

## Results

### Traits Indicating Involvement of Ethylene in Plant Responses to Cd Stress

**FIGURE 1:**
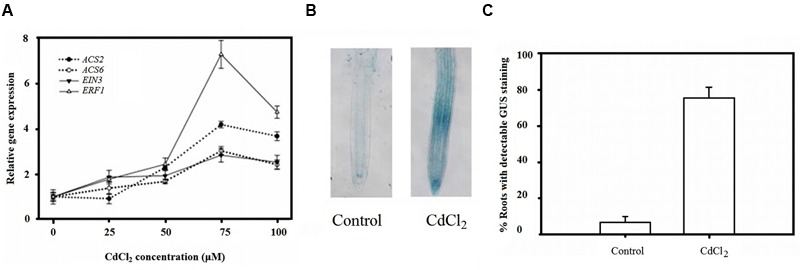
**Ethylene signaling involved in plant root response to Cd stress. (A)** QRT-PCR analysis of relative gene expression of *ACS2, ACS6, EIN3* and *ERF2* in Col-0 plants under Cd stress. **(B)** Increased ethylene response after Cd treatment visualized by EBS::GUS staining. **(C)** Frequency of stained root tip as **(B)** indicated. Seeds were germinated and grown on MS agar plates for 4 days and then transferred to CdCl_2_ (μM) pretreated plates, as indicated, for another 4 days.

To investigate whether ethylene biosynthesis and signaling were involved in the plant response to Cd stress, quantitative RT-PCR was performed using a Real Master Mix kit (Tiangen, Beijing, China) to measure the relative expression of genes encoding for ethylene biosynthesis, and of perception and signaling proteins. The relative expression of *ACS2, ACS6, ERF1*, and *EIN3* increased slightly under small concentrations of Cd but increased drastically after increasing Cd concentrations to 75 μM but decreased with further increase to 100 μM although expression still higher than the control (**Figure 1A**).

To investigate the mechanism by which CdCl2 affects the synthesis and distribution of ethylene, the relative expression of the ethylene reporter construct, EBS::GUS, in which the GUS reporter gene is driven by a synthetic EIN3-responsive promoter, was tested. Remarkably, less than 10% of root tips in unstressed seedlings exhibited GUS staining while nearly 80% of them in stressed seedlings were stained (**Figures 1B,C**). Taken these results together, suggested the involvement of ethylene signaling in plant responses to Cd stress.

### Effect of Cd Stress on Root System Development

The primary root lengths of the wild-type (Col-0) and the *ein2-5* and *ein3-1eil1-1* plants were severely inhibited by 75 μM CdCl_2_ (Supplementary Figure S1).

**FIGURE 2:**
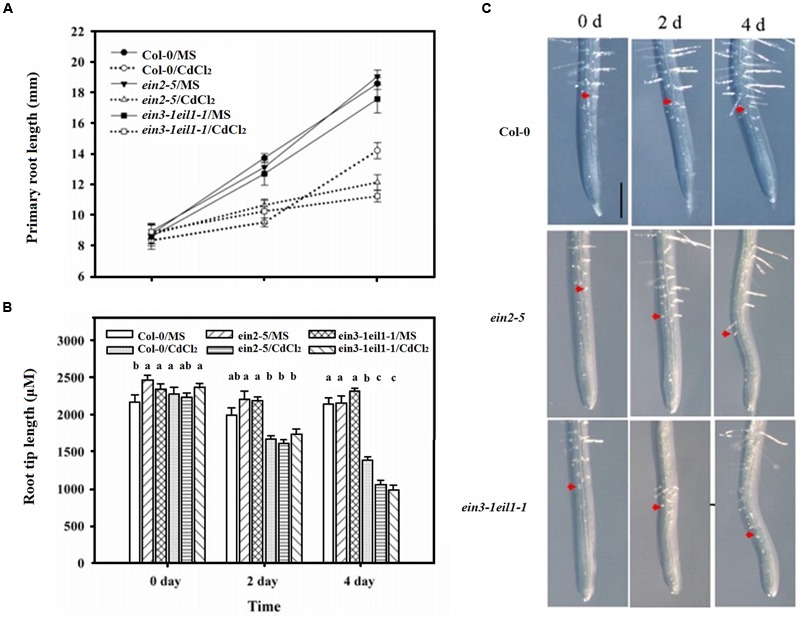
**The inhibitory effects of increased duration of exposure to Cd on the elongation of the primary roots and root tips of the Col-0, *ein2-5*, and *ein3-1eil1-1* plants.** Four-day-old seedlings were transferred to CdCl_2_ (75 μM)-treated agar plates for 2 and 4 days, as indicated. **(A)** The primary root lengths of the Col-0, *ein2-5*, and *ein3-1eil1-1* plants after being transferred to the Cd-treated agar plates for different periods, **(B)** and **(C)** the root tip length. Values represent the mean ± SE of 20 individual plants, and the letters indicate significant differences (*P <* 0.05). Scale bars = 50 μm.

However, **Figure 2A** showed that the primary roots lengths of both the *ein2-5* and *ein3-1eil1-1* plants were slightly longer than those of the wild-type (Col-0) plants after 2 days of Cd stress. This phenomenon was reversed after 4 days treatment as the primary roots of the *ein2-5* and *ein3-1eil1-1* plants were significantly shorter than the wild-type plants.

The root tip length (distance to the first root hair) was also determined after 4 days of Cd treatment (**Figures 2B,C**). Similar to the results for the primary root length, the length of the meristematic and elongation/transition zone was less inhibited in the *ein2-5* and *ein3-1eil1-1* mutants than in the wild-type plants after 2 days of Cd treatment. The length of the zones in the wild-type plant Col-0, *ein2-5*, and *ein3-1eil1-1* plants was 1433.1, 1614.6, and 1741.7 μm, respectively. In contrast, after 4 days treatment, the root tip lengths of both the *ein2-5* (1056.6 μm) and *ein3-1eil1-1* (983.8 μm) plants were significantly shorter compared with the Col-0 (1393.1 μm) plants.

**FIGURE 3:**
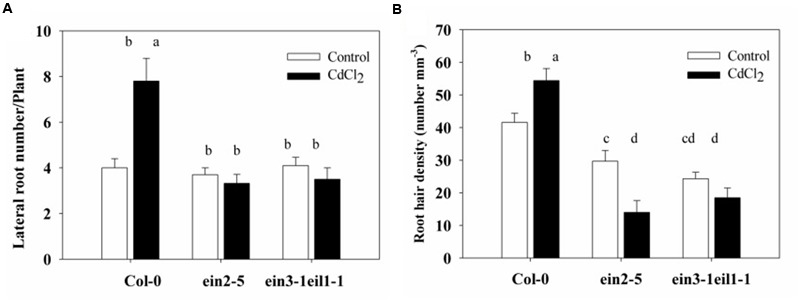
**Effects of Cd stress on the root system architecture of the Col-0, *ein2-5*, and *ein3-1eil1-1* plants. (A)** Lateral root number and **(B)** Root hair density of the 4-days-old seedlings transferred to agar plates with or without CdCl_2_ (75 μM) for 7 days. Values represent the mean ± SE of more than 20 individual seedlings, and the bars indicate the standard error. The letters indicate significant differences (*P <* 0.05).

The lateral root number of the wild-type plants also significantly increased, whereas it was reduced in the *ein2-5* and *ein3-1eil1-1* mutants in response to Cd stress (**Figure 3A**). This result was expected since the primary root of wild-type plants was longer than that of the *ein2-5* and *ein3-1eil1-1* mutants under of Cd stress.

Moreover, the root hair density also significantly increased in the Col-0 plants, whereas it severely decreased in both the *ein2-5* and *ein3-1eil1-1* plants under Cd stress (**Figure 3B**). In addition, it is worth noting that very few root hairs emerged from mutants under Cd stress and also the length of these hairs decreased (Supplementary Figure S2).

These results show that the wild type plants improve their root system development under Cd stress in a better way than *ein2-5* and *ein3-1eil1-1* plants, this led us to suggest the involvement of ethylene in modulating the root system development in response to Cd stress.

### Cd Contents

**FIGURE 4:**
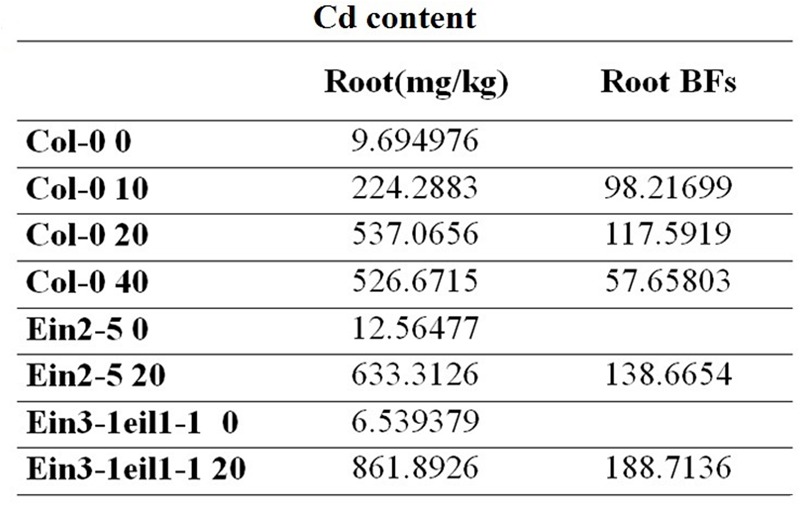
**The Cd content of *Arabidopsis* seedlings roots.** Three-week-old *Arabidopsis thaliana* seedlings were exposed to 0, 10, 20, and 40 μM CdCl2 for 8 days. At the 20 μM CdCl2 treatment, the Cd contents of ein2-5 and ein3-1eil1-1 were even higher than the Cd content of Col-0. Furthermore, the Bfs of ein2-5 and ein3-1eil1-1 were even bigger than Col-0 under 20 μM CdCl2 treatment.

Three-week-old *A. thaliana* seedlings were exposed to 0, 10, 20, and 40 μM CdCl2 for 8 days. (**Figure 4**) showed that a significant accumulation of Cd content in roots treated with Cd. In the Col-0 root, the Cd content was highest-level after exposure to 20 μM CdCl2. At the 20 μM CdCl2 treatment, the Cd contents of *ein2-5* and *ein3-1eil1-1* were even higher than the Cd content of Col-0. Bioaccumulation factors (Bfs) in the Col-0 got their maximum at 20 μM CdCl2. Furthermore, the Bfs of ein2-5 and *ein3-1eil1-1* were even bigger than Col-0 under 20 μM CdCl2 treatment. Ein2-5 and *ein3-1eil1-1* are both ethylene insensitive mutants. Moreover, application of ACC with different concentrations of Cd (50, 60, and 75 μM) decreased the Cd content in roots compared with roots treated with Cd alone (Supplementary Figure S3).

These results showed that ethylene could play a role in plant response to Cd stress supporting the above mentioned results and led us to wonder about the mechanism by which ethylene modulates root system in response to Cd stress.

### Accumulation of Superoxide in Root Tips under Cd Stress

**FIGURE 5:**
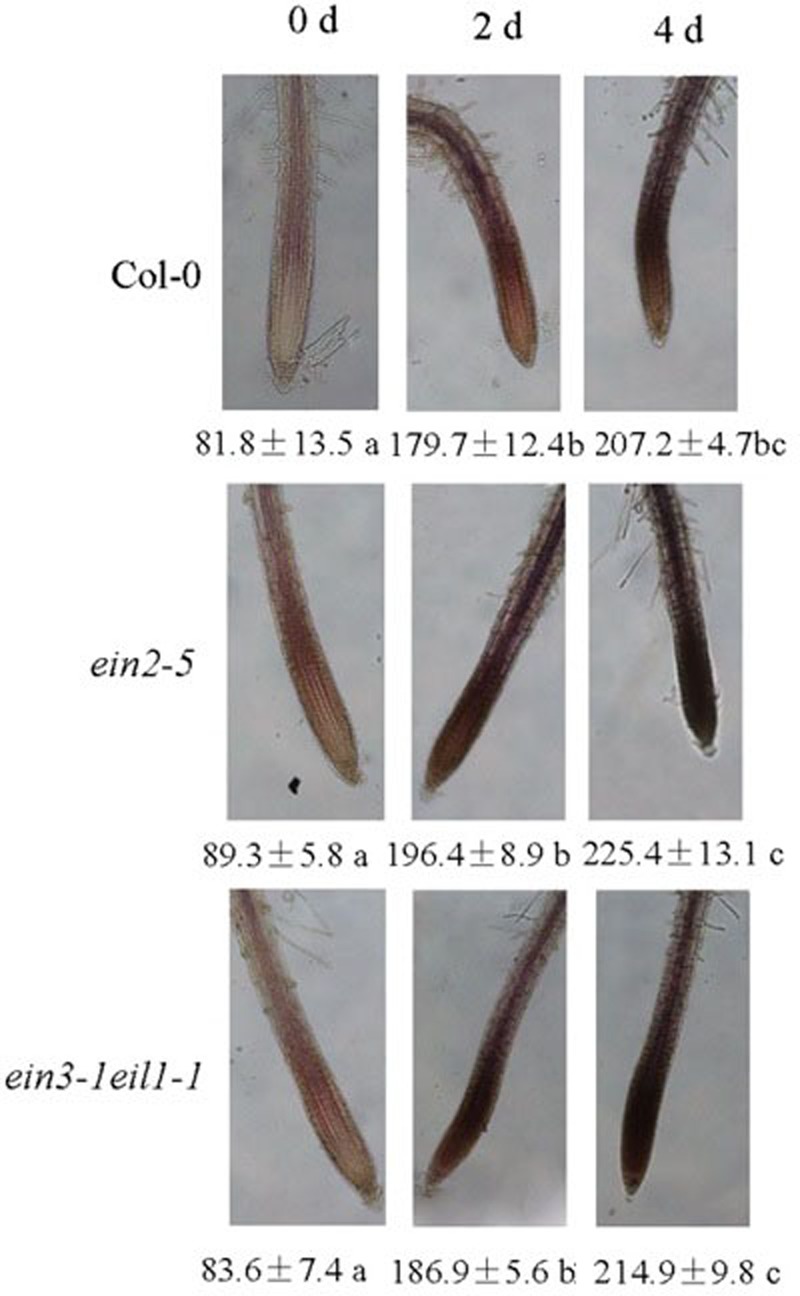
**The accumulation of superoxide in the root tips of the Col-0, *ein2-5*, and *ein3-1eil1-1* plants.** Four-day-old seedlings were transferred to CdCl_2_ (75 μM) -treated agar plates for different periods, as indicated. 2 days treatment significantly increased the production of superoxide in all plants. In addition, the accumulation of superoxide in both the *ein2-5* and *ein3-1eil1-1* mutants was significantly greater than in the wild-type plants even after 4 days of Cd treatment. Values (mean ± SE) show the staining intensity, and the letters indicate significant differences (*P <* 0.05). At least 20 individual roots were analyzed for each genotype and treatment, and one representative image was selected for the figure.

To understand the mechanism of ethylene response to Cd stress, we determined the superoxide in the root tips of Col-0, ein2-5, and *ein3-1eil1-1* under Cd stress. As the results in **Figure 5** indicate, superoxide was predominantly localized at the meristematic and elongation zones. Treatment of the 4-days-old seedlings with 75 μM Cd for 2 days significantly increased the production of superoxide in all plants. However, the accumulation of superoxide in both the *ein2-5* and *ein3-1eil1-1* mutants was significantly greater than in the wild-type plants. Furthermore, after 4 days of Cd treatment, the concentration of superoxide in the root tips was still higher in the two ethylene-insensitive mutants than in the wild-type plants.

The altered response pattern of superoxide accumulation in *ein2-5* and *ein3-1eil1-1* suggested that ethylene signaling regulates superoxide accumulation in the Cd-stressed root tips.

### Effect of ACC (an Ethylene Precursor) on Superoxide Accumulation and Root System Development under Cd Stress

**FIGURE 6:**
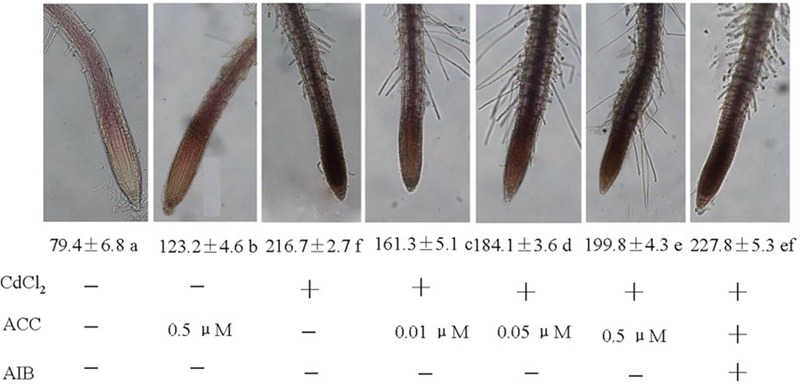
**Effects of exogenous ACC on the accumulation of superoxide in the root tips of the Col-0 plants under Cd stress.** Four-day-old seedlings were transferred to various pretreated agar plates with CdCl_2_ (75 μM) or CdCl_2_ (75 μM) plus various concentrations of ACC (0.01, 0.05, and 0.5 μM, a precursor of ethylene biosynthesis) with or without 5 μM AIB (an inhibitor of ethylene biosynthesis), as indicated, for 4 days. Values (mean ± SE) show the staining intensity, and the letters indicate significant differences (*P <* 0.05). At least 20 individual roots were analyzed for each genotype and treatment, and one representative image was selected for the figure.

To investigate whether regulating superoxide is the mechanism by which ethylene modulate root system under Cd stress, effect of exogenous ACC (a precursor of ethylene biosynthesis) on the accumulation of superoxide in the root tips of the Col-0 plants under Cd stress was determined. Generally, Cd stress-induced increases in superoxide accumulation were detected after 4 days of treatment in the Col-0 root tips as shown in **Figure 6**. Although the supplementation with ACC slightly increased superoxide accumulation, treatment with ACC together with Cd markedly reduced the production of superoxide compared with the Cd treatment alone in the root tips, especially with 0.01 μM concentration of ACC. Conversely, the ACC-induced suppression of the production of superoxide was reversed by AIB (an inhibitor of ethylene biosynthesis) application with ACC and Cd, confirming the results in **Figure 5** and supporting the involvement of ethylene in regulation of superoxide accumulation in the Cd-stressed root tips.

**FIGURE 7:**
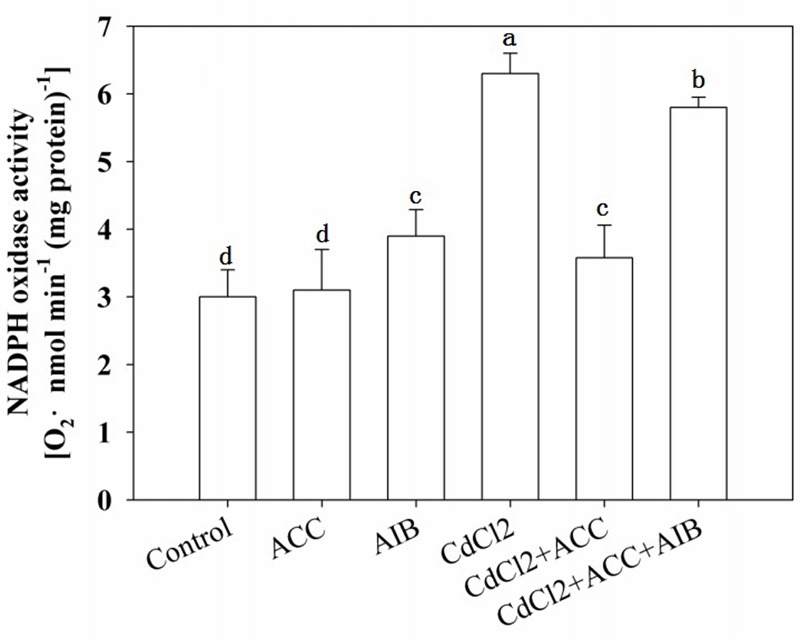
**Effects of various pretreatments (ACC, 0.01 μM; AIB, 5 μM) on the production of O_2_^-^ in roots of *Arabidopsis* Col-0 seedlings under Cd (75 μM) stress.** Four-day-old seedlings were transferred to various pretreated agar plates for 4 days. Values represent the mean ± SE, and the bars indicate the standard error.

It was well established that PMs NADPH oxidase transfers electrons from cytoplasmic NADPH to O_2_ to form O_2_^-^ thus we subsequently determined activity of NADPH in seedlings roots. Same results were also obtained in determining NADPH oxidase activities when exogenous ACC were applied to CdCl2 stressed seedlings and also with the addition of AIB (**Figure 7**).

The effect of ACC on the root system development in Col-0 plants under Cd stress was also determined. Application of exogenous ACC significantly increased lateral roots number and the length of root hairs. The effect of ACC was reversed by AIB that decreased the root hairs density and root hairs length (Supplementary Figure S4).

Taken these results together, we suggested that ethylene modulates root system development under Cd stress by regulating superoxide concentration.

### Effect of Ethylene on SOD and Its Three Isoenzymes Activities in Root under Cd Stress

To investigate whether ethylene reduces Cd stress-induced superoxide accumulation through the SOD pathway, the activities of SOD and its three isoenzymes (Cu/Zn-SOD, Fe-SOD, and Mn-SOD) were measured in Col-0, *ein2-5*, and *ein3-1eil1-1* under Cd stress.

**FIGURE 8:**
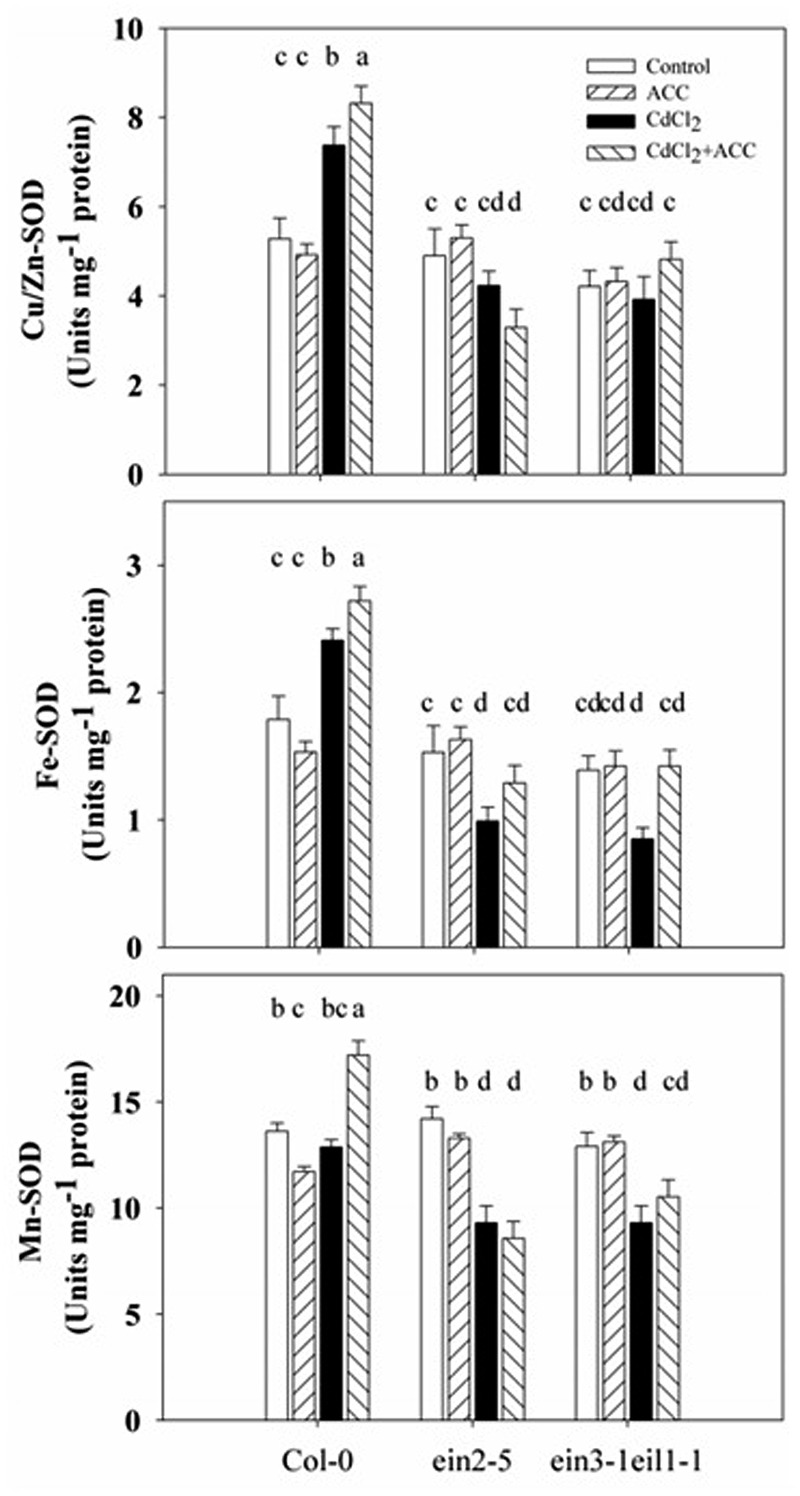
**Activities of SOD and its Cu/Zn-SOD, Fe-SOD, and Mn-SOD isoenzymes in the roots of the Col-0, *ein2-5*, and *ein3-1eil1-1* plants under Cd stress.** Four-day-old seedlings were transferred for 4 days to agar plates, or agar plates with CdCl_2_ (75 μM), or with ACC (0.01 μM, a precursor of ethylene biosynthesis), or with CdCl_2_ (75 μM) plus ACC (0.01 μM), as indicated. Values represent the mean ± SE, and the bars indicate the standard error. The letters indicate significant differences (*P <* 0.05).

As the results in **Figure 8** illustrate, the SOD activities were increased after Cd treatment in the Col-0 but decreased in the *ein2-5* and *ein3-1eil1-1* plants. In addition, the application of exogenous ACC significantly increased these activities compared with the Cd stress in both Col-0 and the *ein3-1eil1-1* mutants. However, ACC displayed no effects on the SOD activities of the *ein2-5* plants. Furthermore, the Cu/Zn-SOD and Fe-SOD activities significantly increased while the Mn-SOD activity did not change after 5 days of Cd treatment in the Col-0. Conversely, the activities of all three isoenzymes decreased in the *ein2-5* and *ein3-1eil1-1* roots. The application of ACC to the Cd-stressed seedlings increased the activities of all three isoenzymes in both the wild-type and *ein3-1eil1-1* plants, whereas ACC only increased the activity of Fe-SOD in the *ein2-5* mutant.

The increased SOD activities in the Col-0 compared with the *ein2-5* and *ein3-1eil1-1* plants under Cd stress suggests that ethylene reduces Cd stress-induced superoxide accumulation through the SOD pathway.

### Effect of Cd Stress on Cell Death in the Meristematic and Elongation Zones

**FIGURE 9:**
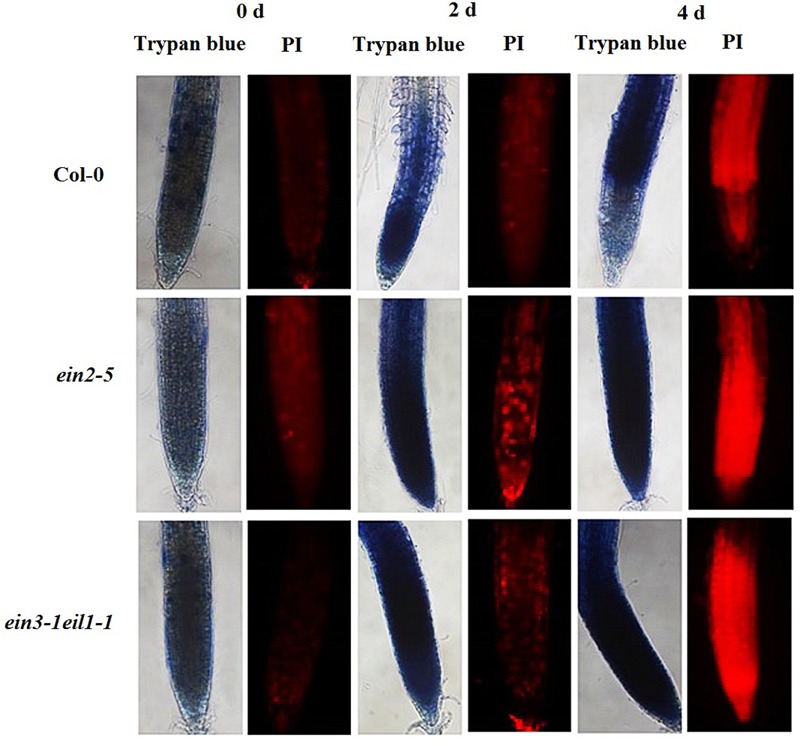
**Progression of cell death/necrosis in the root tips of the Col-0, *ein2-5*, and *ein3-1eil1-1* plants during Cd stress.** Four-day-old seedlings were transferred to CdCl_2_ (75 μM)-treated agar plates for different periods, as indicated. The seedlings were then collected and incubated in propidium iodide (PI) or Trypan blue. At least 20 individual roots were analyzed for each genotype and treatment, and one representative image was selected for the figure.

High concentrations of superoxide induce oxidative stress, which ultimately leads to cell death. We evaluated the progression of cell death in the root tips using PI and Trypan blue. As the results of the PI staining show, exposing the seedlings to Cd stress generally led to significant cell death in the primary root tips after treatment, especially from the end of the meristematic zone to the elongation zone. However, the PI-positive cells staining intensity were significantly increased in the *ein2-5* and *ein3-1eil1-1* mutant root tips compared with the wild-type plants after 2 days of Cd treatment. Furthermore, after 4 days of Cd treatment, the entire meristematic and most of the elongation zone of the stressed root tips were strongly stained in the *ein2-5* and *ein3-1eil1-1* mutants, whereas only the end of the elongation zone was affected in the wild-type plants. Staining analyses using Trypan blue showed a similar progression of cell death, confirming the above mentioned results (**Figure 9**).

### TUNEL and DAPI Assay

**FIGURE 10:**
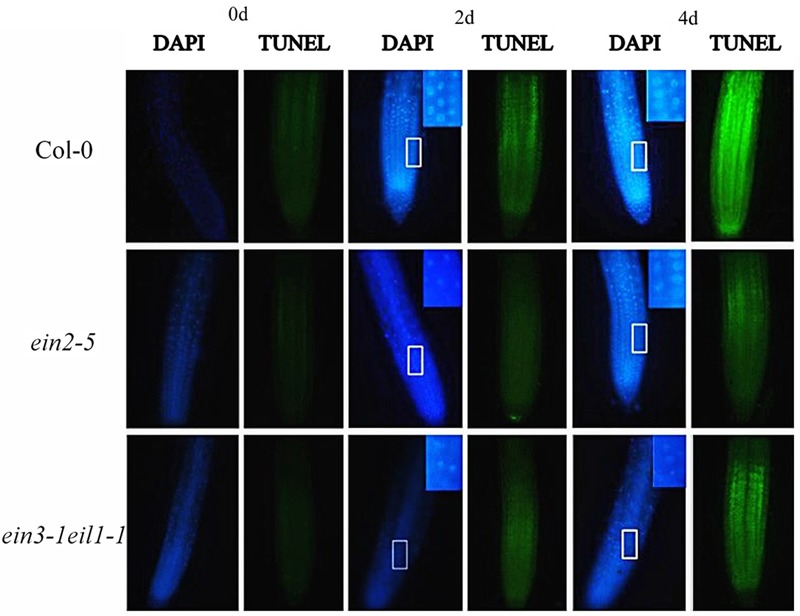
**Progression of programmed cell death, indicated by DAPI and TUNEL staining in the root tips of the Col-0, *ein2-5*, and *ein3-1eil1-1* plants during Cd stress.** Four-day-old seedlings were transferred to CdCl_2_ (75 μM)-treated agar plates for different time points, as indicated. Insets: close up observations enlargement of chromatin condensation. At least 20 individual roots were analyzed for each genotype and treatment, and one representative image was selected for the figure.

As shown in **Figure 10**, only weak DAPI (weak fluorescence and round, homogenously stained nuclei) and TUNEL signals were detected in the untreated seedling roots. However, a marked increase in DAPI fluorescence and condensed and granular nuclear staining and TUNEL-positive signals were detected in the meristematic zone to the elongation zone from root tips after Cd treatment. However, higher intensity of DAPI fluorescence and TUNEL-positive signals were also detected in the wild-type root tips compared with the *ein2-5* and *ein3-1eil1-1* mutants.

## Discussion

In plants, the root system is the first part of plant to suffer from heavy metal toxicity and respond to it (Li et al., 2012; Duan et al., 2015). Thus, the root system is of significant importance and relevance when investigating the responsive and adaptive patterns of plants to environmental stress. This study is particularly to understand the mechanisms by which Cd affects the root system growth and how plants response to this kind of stress. A key feature of the effects of Cd is the increased biosynthesis of ethylene and production of superoxide in roots of *A. thaliana* seedlings. Ethylene was demonstrated to control superoxide concentrations by modulating the activities of SOD isoenzymes.

**Figure 1A** indicated that the expression of both *ACS2* and *ACS6*, which encode two ACS isoforms (ACS2 and ACS6) can be induced by Cd, as observed for other abiotic factors (Smith and Arteca, 2000; Wang et al., 2002; Schellingen et al., 2014). Furthermore, under Cd stress, the expression of *EIN3*, whose expression increases in the presence of ethylene, and the ethylene-responsive gene *ERF1*, which is an immediate target of EIN3 (Solano et al., 1998) were increased, indicating the presence of ethylene in the seedling roots. This confirms that ethylene signaling was involved in the plant response to Cd stress in the roots. However, expression of genes related to ethylene biosynthesis and perception reaches its maximum value at a certain dose of CdCl_2_ (75 μM) but is decreased with a higher concentration (100 μM), indicating that at 100 μM, cells suffered from extensive damage and many cellular components are affected. This would disrupt signaling networks. Moreover, the enhanced expression of these genes might account for the observed Cd-induced stimulation of EBS::GUS activity in the root apex (**Figures 1B,C**). The present results are consistent with a previous study indicating that Cd stress induces rapid ethylene production in root tips (Arteca and Arteca, 2007), suggesting that ethylene is involved in regulating plant responses to heavy metals.

The primary root lengths of the wild-type (Col-0) and the *ein2-5* and *ein3-1eil1-1* plants were severely inhibited by 75 μM CdCl_2_ (Supplementary Figure S1), consistent with a previous report that primary root length decreases in a Cd dose-dependent manner (Schellingen et al., 2015).

However, **Figure 2** showed that the primary roots and the root tip lengths of both the *ein2-5* and *ein3-1eil1-1* plants were slightly longer than those of the wild-type (Col-0) plants after 2 days of Cd stress. This phenomenon was reversed after 4 days treatment as *ein2-5* and *ein3-1eil1-1* plants primary roots and root tips were significantly shorter than those of the wild-type plants.

The lateral root number and the root hairs density of the wild-type plants also significantly increased, consistent with the stress-induced morphogenic response (SIMR) reported before (Bochicchio et al., 2015). Whereas they were reduced in the *ein2-5* and *ein3-1eil1-1* mutants in response to Cd stress (**Figure 3**). Several studies indicate that morphological alterations that result in increased root surface area, such as the formation of root hairs and lateral roots, could be functionally related to stress avoidance mechanisms (Sofo et al., 2013; Vitti et al., 2013).

All of these facts highlighted the essential role of ethylene in the process. It is known that ethylene upregulates auxin biosynthesis in *Arabidopsis* seedlings root tips (Swarup et al., 2007; He et al., 2011), while auxin is involved in lateral root initiation and emergence in a number of plants including *Arabidopsis* (Aloni et al., 2006; Zolla et al., 2010; Sofo et al., 2013). Thus, the crosstalk between ethylene and axuin in regulating lateral root initiation and emergence may also contribute to root SIMR under Cd stress.

**Figure 4** in consistent with a previous study; showed a significant accumulation of Cd content in root treated with Cd (Khan et al., 2016). The Cd contents of *ein2-5* and *ein3-1eil1-1* were even higher than the Cd content of the wild-type. Moreover, the bioaccumulation factors (Bfs) were even bigger in ein2-5 and *ein3-1eil1-1* than Col-0. Application of ACC with different concentrations of Cd (50, 60, and 75 μM) decreased the Cd content in roots compared with roots treated with Cd alone (Supplementary Figure S3). These results suggested that ethylene could play a role in plant response to Cd stress.

The tip of roots is a zone of active ROS production (Liszkay et al., 2004). ROS such as O_2_^-^, H_2_O_2_ and HO^∙^ are considered as key factors in the oxidative burst, and play important physiological roles in plants (Mittler et al., 2004). Cd stress is related to the increase of ethylene and ROS (Xu et al., 2010a). The oxidative damage of ROS requires the action of antioxidative enzymes, including SOD, which can convert superoxide radicals into hydrogen peroxide, water and oxygen. As the results indicate, Cd stimulated superoxide production in the root tips (**Figure 5**), consistent with the results of Liptáková et al. (2012). However, the accumulation of superoxide in both the *ein2-5* and *ein3-1eil1-1* mutants was significantly greater than in the wild-type plants. The altered response pattern of superoxide accumulation in *ein2-5* and *ein3-1eil1-1* suggested that ethylene signaling regulates superoxide accumulation in the Cd-stressed root tips.

Application of exogenous ACC suppressed Cd stress-induced production of superoxide in the root tips of wild type plants (**Figure 6**), consistent with the salinity stress results (Lin et al., 2013a). On the other side, application of AIB, the inhibitor of ethylene biosynthesis, together with ACC and Cd reversed the ACC-induced suppression of superoxide production, consistent with the reverse effect of AIB to ACC effect on seed germination under stress (Lin et al., 2013b) and depending on the fact that AIB inhibits the endogenous as well as the ACC-dependent ethylene production (Satoh and Esashi, 1982), these results supports the involvement of ethylene in regulation of superoxide accumulation in the Cd-stressed root tips.

In agreement with former results, CdCl_2_ treatment increased NADPH oxidase activity while application of exogenous ACC decreased CdCl_2_ induced NADPH oxidase activity. Moreover, application of AIB with ACC and Cd reversed the ACC effect and increased NADPH oxidase activity (**Figure 7**), indicating ethylene regulation of O_2_^∙-^ production under Cd stress, consistent with pervious result that an oxidative burst induction by NADPH oxidases under stress is connected to ethylene (Montero-Palmero et al., 2014).

In accompanied to the suppressed Cd stress-induced production of superoxide and the decreased CdCl_2_ induced NADPH oxidase activity, roots hairs density and root hairs length increased under CdCl_2_ stress by application of exogenous ACC (Supplementary Figure S4). The effect of ACC (a precursor of ethylene biosynthesis) was reversed by AIB (an inhibitor of ethylene biosynthesis). Taken these results together, we suggested that ethylene modulates root system development under Cd stress by regulating superoxide concentration.

Interestingly, under Cd stress, the activities of all three SOD isoenzymes increased in the wild-type plants compared with *ein2-5* and *ein2-5eil1-1*. Moreover, application of exogenous ACC to the Cd-treated seedlings increased all three SOD isoenzymes activities in the wild-type plants (**Figure 8**), consistent with the increased SOD activity in Col-0 plants that was accompanied by a significant up-regulation of the genes *FeSOD* compared with *ein2-5* mutant under salt stress (Lin et al., 2013a). Results in Supplementary Figure S5 show up-regulation of the three SOD genes in Col-0 plants compared with *ein2-5* mutant under oxidative stress induced by salinity. This observation that suggests that ethylene may directly increase SOD activity under Cd stress, may be the underlying mechanism through which ethylene controls the amount of superoxide, which then initiates distinct forms of responses and acclimations depending upon the levels of superoxide in *Arabidopsis* root tips under Cd stress.

High concentrations of superoxide lead to oxidative stress, which directly inhibits or modifies some proteins and ultimately induces cell death (Miller et al., 2010). There are two types of cell death, necrosis and apoptosis, which is a subset of PCD. PI, a nucleic acid dye that intercalates into double-stranded nucleic acids, is excluded from viable cells but can penetrate the cell membranes of dead cells (Ning et al., 2002; Duan et al., 2010). Generally, Cd treatment induced the onset of necrosis at the meristematic and elongation zones of all plants compared with the control (**Figure 9**). However, greater PI-positive staining was observed after (4 days) Cd treatment in both the *ein2-5* and *ein3-1eil1-1* mutant root tips compared with the Col-0 plants. In addition, these results were confirmed by Trypan blue staining, another molecular prove for cell necrosis. These results suggest that ethylene may play crucial roles in preventing cells of the meristematic and elongation zones from undergoing necrosis during stress and recovery periods when plants are facing stresses.

Programmed cell death is an activate process of cellular suicide that is essential for development and stress responses in plants. To examine whether PCD is involved in cell death at root tips, DAPI staining and the TUNEL assay, were used, respectively, which are typically used as diagnostic markers for PCD (Duan et al., 2010; Xu et al., 2010b). In contrast to the progression of necrosis, the initiation of PCD was more rapid and intensive in the wild-type plant root tips compared with the *ein2-5* and *ein3-1eil1-1* plants, confirming the critical role of ethylene in the progression of PCD under Cd stress (**Figure 10**).

**FIGURE 11:**
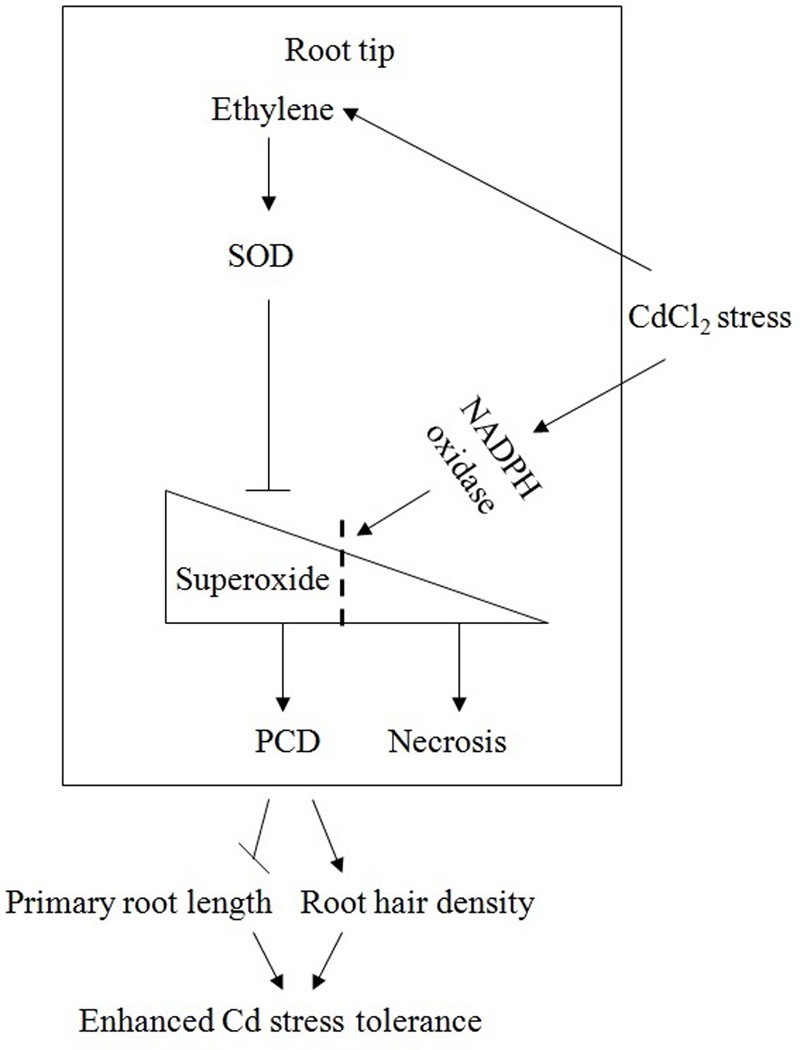
**A schematic model elucidating the interaction of ethylene, SOD, superoxide and PCD in the root meristematic and elongation zones under Cd stress**.

Based on our combined results, a schematic model (**Figure 11**) elucidating the interaction of ethylene, SOD, superoxide and PCD in the root meristematic and elongation zones under Cd stress, is presented. In this model, Cd treatment induces the rapid production of ethylene and superoxide by the gene up-regulation of ethylene synthesis and the Cd-caused oxidative damage, respectively. Under Cd stress, the presence of ethylene maintains or increases the activity of SOD, which maintains the level of superoxide lower than what expected without ethylene and consequently prevent cells of the meristematic and elongation zones from undergoing necrosis. Superoxide could function as a signaling molecule that initiates the occurrence of PCD in root tips at the early stage of Cd. PCD occurs in the region of root apical meristem in undifferentiated cells, where it alters root apical dominance and remodels the root system architecture, inhibiting primary root elongation and increasing lateral roots number and root hairs density, perhaps to minimize the damage caused by stress conditions. This response is an adaptive and acclimation strategy of plants to adverse environments.

## Conclusion

The presence of ethylene enables plants to perceive adverse stimuli and to respond effectively, thus enhancing their acclimation and adaptation to stress. In *ein2-5* and *ein3-1eil1-1* seedlings, the increased superoxide concentration induced by Cd stress resulted in cell death and defects in root growth and development in the root meristem zone, by which root elongation was inhibited. In contrast, ethylene reduced the concentration of superoxide maintained in wild type plants. Moreover, superoxide concentration was reduced by ACC application and reversely increased by application of AIB that inhibits the endogenous as well as the ACC-dependent ethylene production. The ethylene-induced suppression of superoxide decreased the occurrence of cells death and initiated PCD to minimize the damage caused by Cd stress. Hence, this is a general adaptive mechanism in *A. thaliana* through ethylene induction to improve root system development by modulating superoxide anion concentration under Cd stress.

## Author Contributions

ZT conceived and designed the experiments. AA, ZY, and YL performed the experiments and wrote the manuscript. JL and ZZ revised the manuscript. All authors reviewed the manuscript.

## Conflict of Interest Statement

The authors declare that the research was conducted in the absence of any commercial or financial relationships that could be construed as a potential conflict of interest.

The reviewer MA and handling Editor declared their shared affiliation, and the handling Editor states that the process nevertheless met the standards of a fair and objective review.
