# Supplementary material for: Ethylene Improves Root System Development under Cadmium Stress by Modulating Superoxide Anion Concentration in Arabidopsis thaliana
Source: Front Plant Sci. 2017 Feb 24;8:253. doi: 10.3389/fpls.2017.00253 (PMC5323375; doi:10.3389/fpls.2017.00253)
Supplement: Supplementary file 1 [file Data_Sheet_1.DOCX]

Supplementary Material

**Ethylene improves root system development under cadmium** **stress by modulating superoxide anion concentration in *Arabidopsis* *thaliana***

Ann Abozeid, Zuojia Ying, Yingchao Lin, Jia Liu, Zhonghua Zhang, Zhonghua Tang*

*** Correspondence:** Corresponding Author: [tangzh@nefu.edu.cn](mailto:tangzh@nefu.edu.cn)

**
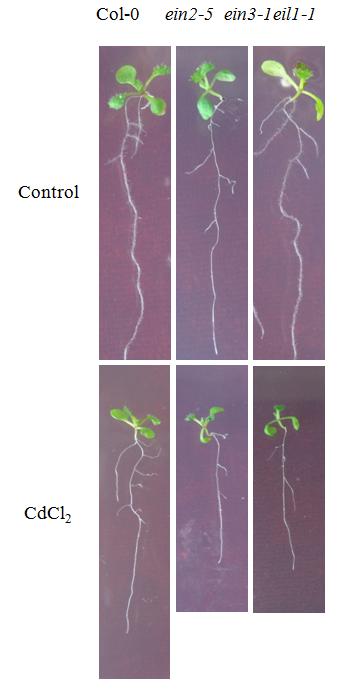
Supplementary Figure S1**

**Supplementary Figure S1.** Response of root elongation in *Arabidopsis* wild-type (Col-0), *ein2-5* and *ein3-1eil1-1* seedlings to CdCl_2_. The lengths of primary roots were measured after the 4-d-old seedlings were cultivated on agar plates containing CdCl_2_ (75 μM) for 4 d. The primary root lengths of all plants were severely inhibited by CdCl_2_ (75 μM)_._

**
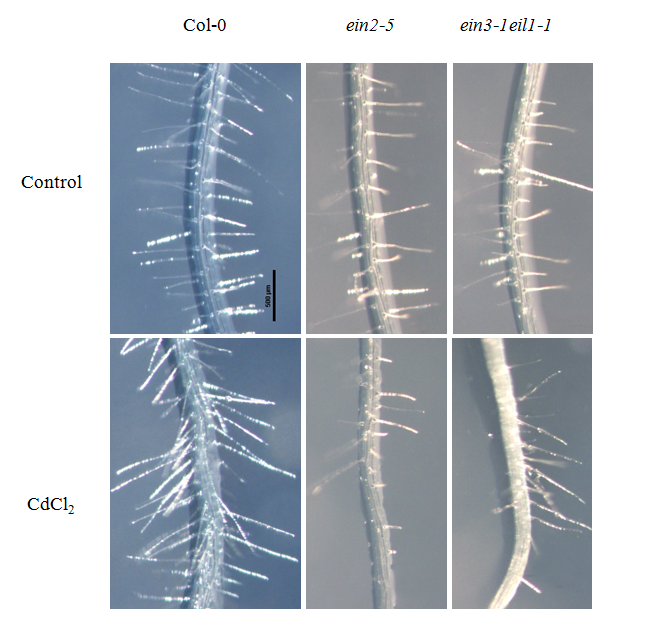

Supplementary Figure S2**

**Supplementary Figure S2.** Distribution of the root hair length for Col-0, *ein2-5* and *ein3-1eil1-1* seedlings. Four-day-old seedlings were transferred to agar plates with or without CdCl_2_ (75 μM)for 7 d. At least 200 root hairs were measured for each genotype.

**
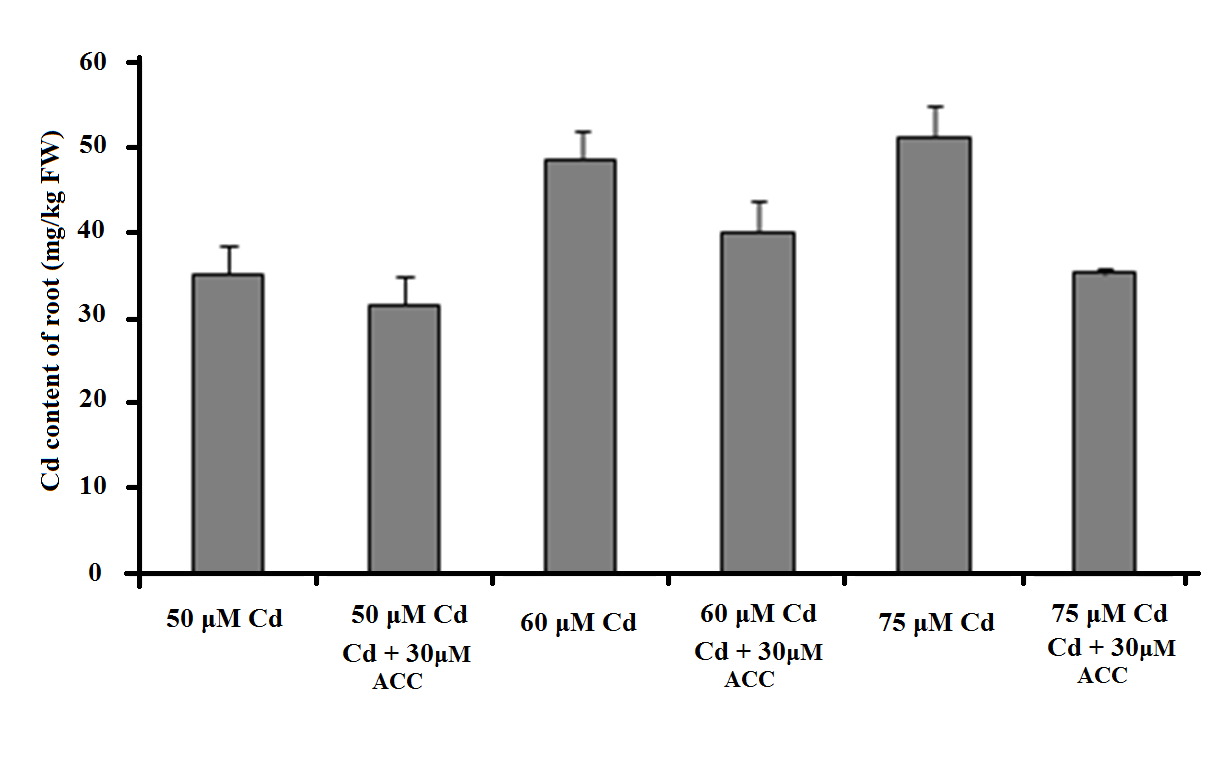
**

**Supplementary Figure S3**

**Supplementary Figure S3.** The effect of ethylene on Cd content of *Arabidopsis thaliana* seedling under Cd stress. Four-day-old *Arabidopsis* seedlings were exposed to 50, 60 and 75 µM CdCl2, with or without 30 µM ACC for 14 days. Compared with seedlings treated with Cd alone, the content of Cd in roots treated with 30μM AAC had decreased. Under Cd stress the ethylene could reduce the Cd content of *Arabidopsis* seedlings.

**
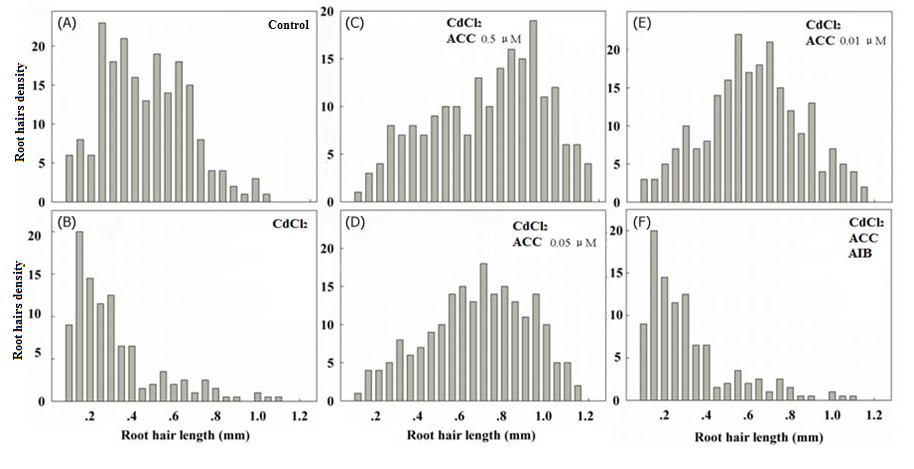
**

**Supplementary Figure S4**

**Supplementary Figure S4.** Response of root hair density and the root hairs length to the presence of ACC and AIB in *Arabidopsis* wild-type (Col-0) under CdCl_2_ stress. Four-day-old seedlings were transferred to agar plates as control (A), agar plates pretreated with CdCl_2_ (75 μM) (B), or CdCl_2_ (75 μM) plus various concentrations of ACC (0.01, 0.05 and 0.5 μM, a precursor of ethylene biosynthesis) (C), (D) and (E); or CdCl_2_ (75 μM) plus 0.01 μM ACC with 5 μM AIB (an inhibitor of ethylene biosynthesis) (F), values are means±SE of >20 roots. The letters indicate significant differences (*P*<0.05). For (D), at least 200 root hairs were measured for each treatment in total.


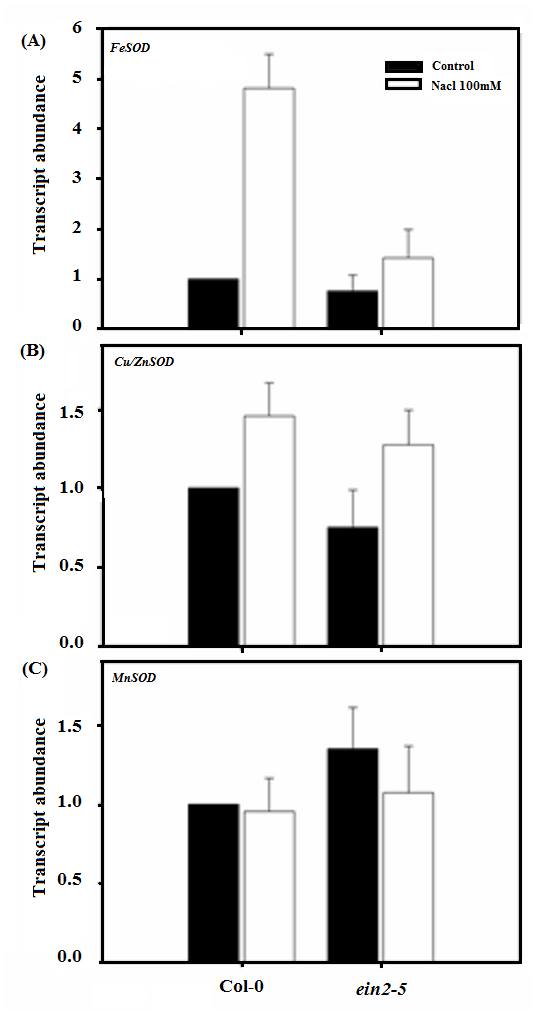
**Supplementary Figure S5**

**Supplementary Figure S5.** Microarray analysis of relative expression of DEFL and three ROS-scavenging genes *FeSOD, Cu/ZnSOD, MnSOD* in Col-0 and *ein2-5* plants germinated and grown on MS agar plates with or without 100 mM NaCl as indicated for 4 days. Values shown are mean ± SE of three biological replicates.
